# A prospective pilot study of detection of sentinel lymph nodes in gynaecological cancers using a novel near infrared fluorescence imaging system

**DOI:** 10.1186/s13104-015-1576-z

**Published:** 2015-10-26

**Authors:** Alexandros Laios, Davide Volpi, Iain D. C. Tullis, Martha Woodward, Stephen Kennedy, Pubudu N. J. Pathiraja, Krishnayan Haldar, Borivoj Vojnovic, Ahmed A. Ahmed

**Affiliations:** Nuffield Department of Obstetrics and Gynaecology, University of Oxford, Oxford, UK; Gynaecological Oncology Unit, Churchill Hospital, Oxford University Hospitals NHS Trust, Oxford, UK; Department of Oncology, CRUK/MRC Oxford Institute for Radiation Oncology, University of Oxford, Oxford, UK; Early Phase Research Hub, Department of Oncology, Oxford Cancer and Haematology Centre, Oxford University Hospitals NHS Trust, Oxford, UK; Weatherall Institute of Molecular Medicine, John Radcliffe Hospital, University of Oxford, Headington, Oxford, OX37DS UK

**Keywords:** Gynaecological cancer, Sentinel lymph node, Near infrared fluorescence, Optical imaging, Indocyanine green, Methylene blue

## Abstract

**Background:**

Sentinel Lymph Node (SLN) sampling may significantly reduce surgical morbidity by avoiding needless radical lymphadenectomy. In gynaecological cancers, the current practice in the UK is testing the accuracy of SLN detection using radioactive isotopes within the context of clinical trials. However, radioactive tracers pose significant logistic problems. We, therefore, conducted a pilot, observational study to assess the feasibility of a novel optical imaging device for SLN detection in gynaecological cancers using near infrared (NIR) fluorescence.

**Methods:**

A novel, custom-made, optical imaging system was developed to enable detection of multiple fluorescence dyes and allow simultaneous bright-field imaging during open surgery and laparoscopic procedures. We then evaluated the performance of the system in a prospective study of 49 women with early stage vulval, cervical and endometrial cancer who were scheduled to undergo complete lymphadenectomy. Clinically approved fluorescent contrast agents indocyanine green (ICG) and methylene blue (MB) were used. The main outcomes of the study included SLN mapping detection rates, false negative rates using the NIR fluorescence technique and safety of the procedures. We also examined the association between injection sites and differential lymphatic drainage in women with endometrial cancer by fluorescence imaging of ICG and MB.

**Results:**

A total of 64 SLNs were detected during both open surgery and laparoscopy. Following dose optimisation and the learning phase, SLN detection rate approached 100 % for all cancer types with no false negatives detected. Fluorescence from ICG and MB detected para-aortic SLNs in women with endometrial cancer following uterine injection. Percutaneous SLN detection was also achieved in most women with vulval cancer. No adverse reactions associated with the use of either dyes were observed.

**Conclusions:**

This study demonstrated the successful clinical application of a novel NIR fluorescence imaging system for SLN detection across different gynaecological cancers. We showcased the first *in human* imaging, during the same procedure, of two fluorescence dyes in women with endometrial cancer.

**Electronic supplementary material:**

The online version of this article (doi:10.1186/s13104-015-1576-z) contains supplementary material, which is available to authorized users.

## Background

The most reliable currently used method for assessing lymph node (LN) status for staging purposes is to perform systematic lymphadenectomy. This is associated with a high degree of morbidity, particularly if adjuvant therapy is administered [1]. Complications include lymphoedema, lymphocyst formation, deep vein thrombosis, associated pressure symptoms and altered sensation in limbs. There is therefore a need for minimally invasive surgical procedures to reduce post-operative morbidities that are associated with systematic lymphadenectomy [2].

The role of sentinel lymph node (SLN) biopsy is an evolving concept in vulval, cervical and endometrial cancers [3]. Conventional methods for detecting SLNs include the use of vital dyes such as isosulphan-blue, patent-V and methylene blue, as well as radioisotope labelled agents [4, 5]. The concurrent use of two techniques produces exceptional detection rates in some cases [6]. However, exposure to ionising radiation and the need for a nuclear medicine unit limit the potential use of the radioactive tracer technique.

Intra-operative near infrared (NIR) fluorescence has emerged as an alternative SLN imaging modality because of its high sensitivity and spatial resolution, as well as good depth penetration [7]. A number of studies in gynaecological cancers using indocyanine green (ICG) dye have shown detection rates comparable to the combined vital-radiotracer method [8–10]. However, other studies reported lower detection rates [11–13]. This discrepancy is likely due to a lack of practice standardisation when different imaging systems of given fluorescence sensitivity and varied injected doses are used. Moreover, the injection practice in endometrial cancers is still questionable, as cervical injection might not be appropriate for all endometrial tumours [14].

To address these issues, we conducted a clinical feasibility study across different cancer types using an in-house developed NIR fluorescence imaging system. The primary objective was to assess the feasibility of using this novel NIR fluorescence imaging system for SLN mapping in vulval, cervical and endometrial cancers during both open and laparoscopic surgery. Secondary objectives included determination of SLN fluorescence detection rates, injected dose optimisation, false negative rates and safety of the procedures. We also aimed to determine the association between injection sites and SLN detection in a subset of women with endometrial cancer by fluorescence imaging of two clinically approved fluorescent dyes.

## Methods

### Clinical trial

PIONIR (An observational PIlot Study Of Near InfraRed imaging of sentinel nodes in early stage vulval, cervical and endometrial cancers using indocyanine green and methylene blue as fluorophores to assess clinical feasibility).

The study was approved by the independent Research Ethics Committee (REC) in Oxforsdshire and the Oxford University Hospital NHS Trust (Ethics Ref: 11/SC/0099) on 03 February 2012 and performed in accordance with the 1975 Helsinki Declaration ethical standards.

### Study design and participants

**Fig. 1:**
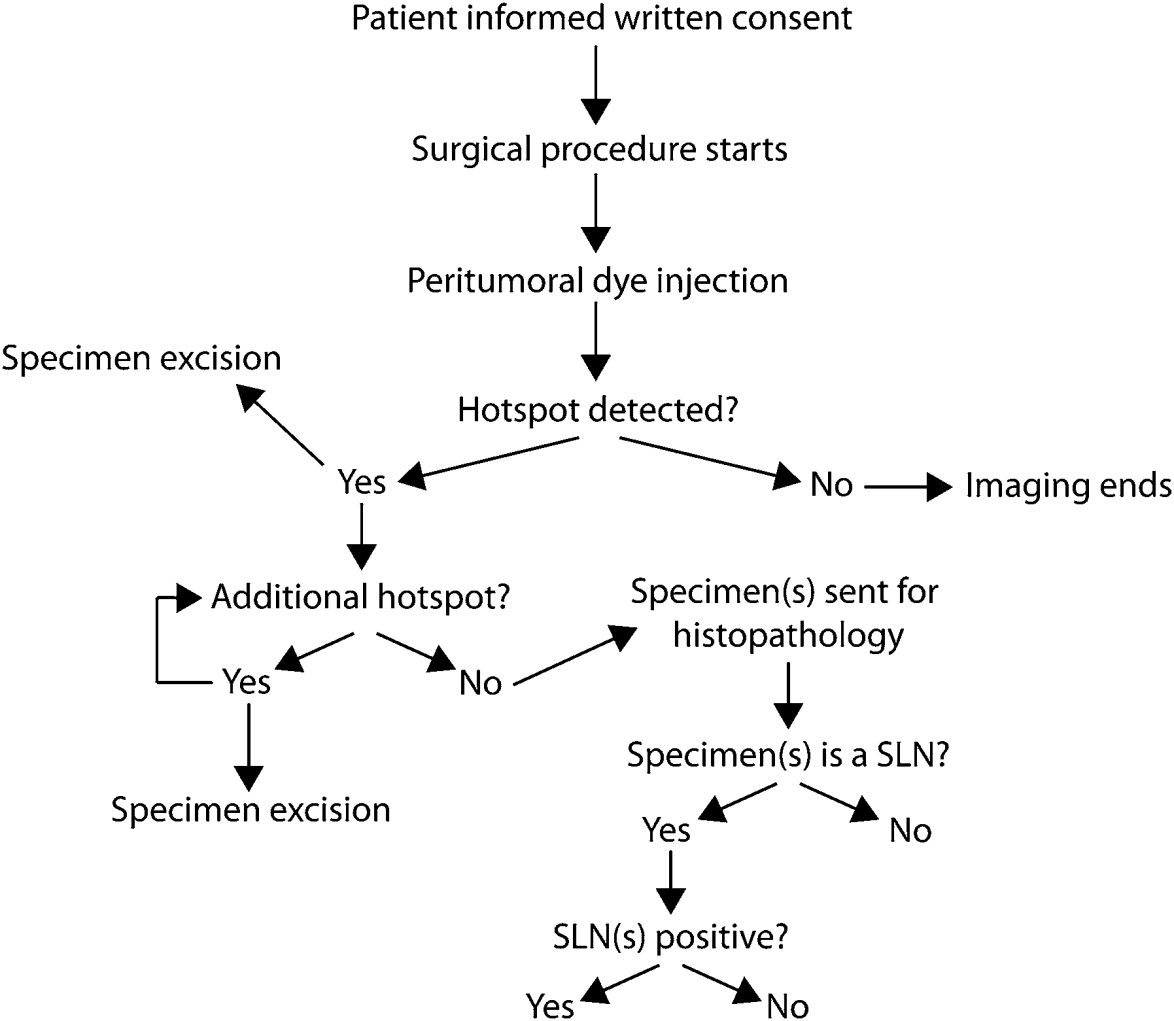
Clinical study flowchart

This pilot study was performed at a tertiary cancer referral centre between October 2012 and September 2014. A total of 49 women with gynaecological malignancies were recruited. Participants had early vulval, cervical and endometrial cancers and were scheduled for complete lymphadenectomy. Exclusion criteria included: (1) age <18 years; (2) pregnancy; (3) allergy to fluorescence dyes; (4) previous chemotherapy, radiotherapy or surgery to the LNs of interest; (5) patient vulnerability and (6) lack of capacity to provide informed consent or unwillingness to inform the family doctor about participation in the study. Demographic and clinical data were collected. Figure 1 shows a schematic decision tree flow-chart of the procedure.

### Fluorescence dyes and injection technique

Clinically approved, NIR fluorescence dyes, indocyanine green (ICG) and methylene blue (MB) were used according to the study protocol. ICG (25 mg vials, Pulsion Medical Systems, Munich, Germany) was re-suspended in sterile water for injection to obtain an initial concentration of 5 mg/ml. The dye was freshly prepared on the day of surgery and one vial was used for each procedure. Following induction of general anaesthesia, 1 ml of ICG was injected immediately before surgery. Dye concentrations and volumes fluctuated during the ‘learning phase’ until an optimal detection rate was achieved. MB (Methylthioninium chloride, Provepharm SAS, Marseille, France) was supplied as ‘Proveblue’, being already dissolved in water to 5 mg/ml. The injected volume was 4 ml. Patients were continuously monitored for side-effects.

For vulval cancer patients, intradermal injection of ICG was performed using a 27G needle, with the needle held at a ~10° angle relative to the skin. Once the bevel pierced the epidermis, it was rotated by 180° before injection at each of four peri-scarring/tumoural margins. In women with cervical cancer, subepithelial ICG injection was performed using a 27G needle into the 3- and 9-o’clock positions, initially submucosally and then deep into the stroma, on both sides of the cervix. In women with endometrial cancer, cervical injection was performed, as described above. In two selected cases, a second dye injection into the uterine fundus (midline subserosa) was performed using a 23G epidural needle. In the first woman, cervical injection of ICG was followed by MB injection in the uterine subserosa. The second woman had reversed dye injection (i.e. MB in the cervix and ICG in the uterine subserosa).

### Intra-operative NIR fluorescence imaging

**Fig. 2:**
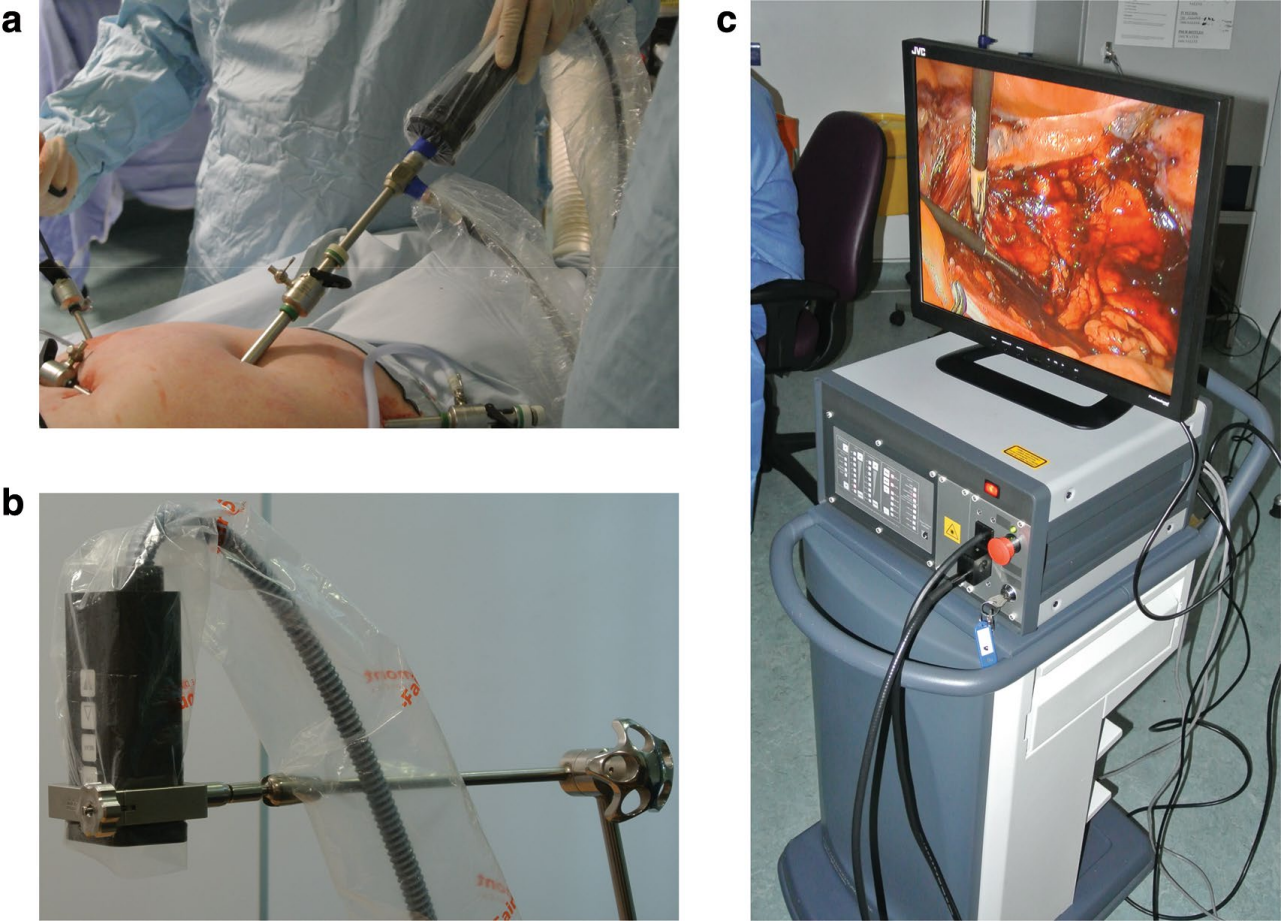
Fluorescence imaging system. **a** Laparoscopic camera attachment, **b** wide-field imaging head and **c** system control unit

A custom-made laparoscopic attachment (Fig. 2a) and a wide-field imaging head (Fig. 2b) (described in [15]) were able to support both laparoscopic and open surgery procedures. Both systems share the same instrument control interface (Fig. 2c) and thus can be rapidly interchanged, if necessary. Two monochromatic (<4 nm bandwidth) sources provided excitation light suitable for ICG and MB fluorescence imaging. A novel optical filtering arrangement allowed real-time simultaneous detection of bright-field colour images and fluorescence images from multiple dyes using a single camera. Fluorescence imaging was performed after dye(s) injection using both wide-field and laparoscopic imagers, as dictated by the surgical procedure. Live video images were displayed on the operating room monitors and the procedures were recorded. Exposure time during fluorescence acquisition ranged between 40 and 320 ms/frame, depending on the emission intensity and imaging depth. In a subset of vulval cancer patients, percutaneous NIR fluorescence imaging was performed suprapubically prior to surgical procedure. The surgeon estimated whether the fluorescence signal was sufficient to warrant resection based on subjective observation. Fluorescence from intra-operative hotspots was verified ex vivo using the same system.

### Surgical procedures

For cervical and endometrial cancer cases, laparoscopic and/or open surgery procedures were performed at the surgeon’s discretion. Pelvic side-walls were opened bilaterally along the external iliac vessels; internal iliac and obturator spaces were exposed prior to inspection with meticulous haemostasis to avoid obscuring lymphatic pathways. In women with vulval cancer, surgical dissection involved systematic exploration of both superficial and deep tissue planes over the femoral triangle and 4-6 cm along the femoral vessels.

### Specimen handling and histopathology

Once removed, the fluorescence hotspots and all the resected LNs during systematic lymphadenectomy were labelled and dispatched for histopathological examination. SLN identification was carried out using standard diagnostic criteria. All LNs were assessed for metastases and their areas were measured. Histopathology was used to determine (1) the presence or absence of a SLN and (2) tumour metastasis in the excised specimens. The pathological protocol involved ultra-staging of SLNs.

### Statistical analysis

The mean age, number of hotspots identified and anatomical location but not site were recorded. The hotspot detection rate was calculated as the ratio between the number of cases in which at least one hotspot was detected and the total number of cases. In line with previous studies, the SLN detection rate was calculated as the ratio between (a) the number of cases in which at least one hotspot was detected and confirmed to be a SLN by histological analysis, and (b) the total number of cases. Successive groups of ten cases were used to determine the learning curve.

The false negative rate was calculated as the number of cases in which at least one LN (non SLN) was positive and the SLN(s) was negative, divided by the total number of cases. The true positive rate was calculated as the number of cases in which at least one fluorescently detected SLN was indeed correctly identified as a cancer positive LN divided by the total number of cases. Data were presented as percent or range (min–max).

## Results

### Characteristics of participants

Forty-nine women with a gynaecological cancer who underwent complete lymphadenectomy were included in this study: 11 (22.4 %) had vulval cancer, 10 (20.4 %) cervical cancer and 28 (57.2 %) endometrial cancer. Their mean age was 61.6 ± 13.2 (range 48–73) years.

### Intra-operative fluorescence detection

**Fig. 3:**
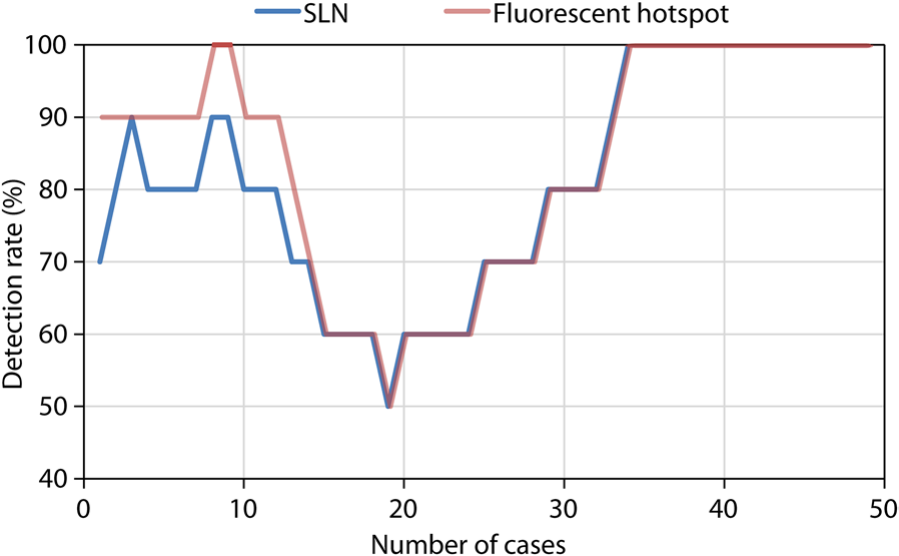
SLN and fluorescence hotspot detection rate showing the learning curve associated with the study

**Fig. 4:**
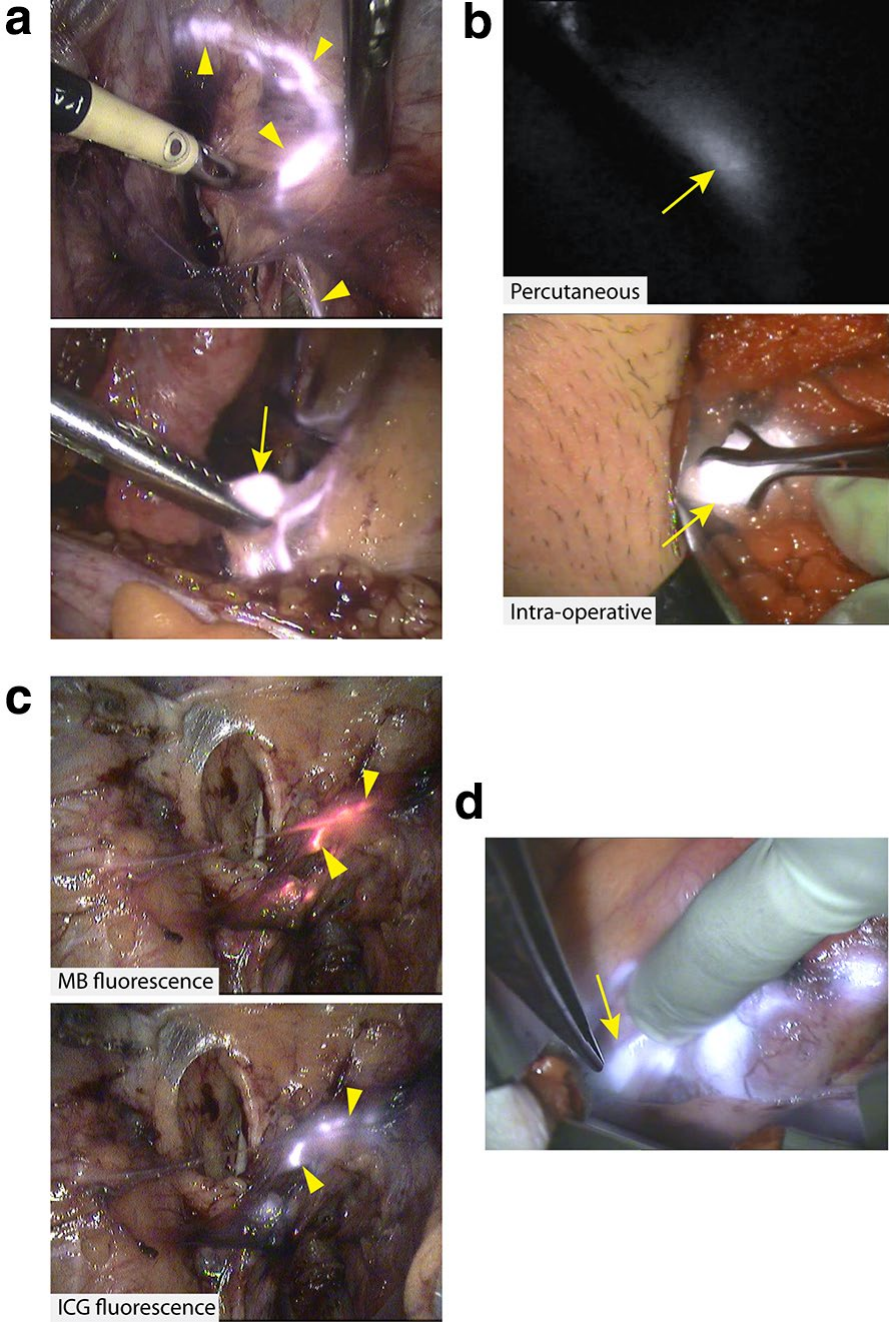
Intra-operative fluorescence imaging. **a** Laparoscopic fluorescence imaging of ICG in a woman with endometrial cancer showing lymphatic vessels (*yellow arrow heads,* *top*) and a SLN (*yellow arrow,* *bottom*). **b** Percutaneous (*top*) and intra-operative (*bottom*) imaging of a ~20 mm deep SLN (*yellow arrow*) in vulval cancer using the wide-field imaging system. **c** Lymphatic co-localisation (*yellow arrow heads) *of MB (*top*) and ICG (*bottom*) following uterine and cervical injection, respectively, in a woman with endometrial cancer. **d** Para-aortic SLN (*yellow arrow*) fluorescence detection in a woman with endometrial cancer following uterine subserosa injection of ICG

Intra-operative hotspot and SLN detection rates are represented by the learning curve shown in Fig. 3. Fluorescence hotspots differed slightly from SLN detection rates at the beginning of the study, indicating that some hotspots contained adipose tissue. Detection rates reached 100 % after ~30 cases, as shown in Table 1, along with total number of SLNs detected. The optimal injected concentration and volume were found to be 1 mg/ml and 4 ml respectively (optimised dose group in Table 1). Considering the optimised dose group only, the average number of SLNs per case was 2 (1–3) for vulval, 4 (4–4) for cervical and 1.5 (1–3) for endometrial cancers. The external iliac group of LNs was the commonest anatomical site for SLN detection for cervical and endometrial cancers. A series of snapshots from fluorescence imaging of lymphatic vessels and a left external iliac SLN in endometrial cancer are shown in Fig. 4a. All intra-operatively identified hotspots were confirmed to be fluorescent by subsequent ex vivo imaging. No residual hotspots were observed at the end of the procedures following systematic inspection of the surgical field. No adverse reactions associated with the use fluorescence dyes were observed.

**Table 1:** Main study outcomes

		All cases (n = 49)	Optimised dose group (n = 16)
SLN detection rate	Vulval	91 %	100 %
	Cervical	90 %	100 %
	Endometrial	68 %	100 %
	Total	78 %	100 %
Average SLNs detected per woman	Vulval	1.1 (0–4)	2 (1–3)
	Cervical	2.3 (0–4)	4 (4–4)
	Endometrial	0.9 (0–3)	1.5 (1–3)
	Total	1.3 (0–4)	1.9 (1–4)

### Histopathological analysis

Metastasis in the excised LNs (including SLNs) was detected in 20.4 % of the cases. The detected SLNs correctly predicted the metastatic state of all the other LNs (no false negative SLNs) when the cases which corresponded to the learning phase and dye optimisation were excluded. The false negatives and true positives rates are shown in Table 2.

**Table 2:** Histopathological analysis results

	All cases (n = 49)	Optimised dose group (n = 16)
False negatives	4 (8 %)	0 (0 %)
True positives	6 (12 %)	2 (12.5 %)

### Percutaneous SLN detection in vulval cancer

Percutaneous detection of inguinal SLNs, prior to surgical incision, was attempted in seven women with vulval cancer using ICG fluorescence wide-field imaging. The fluorescence could be visualised as early as ~6 min post-injection. Groin incision was then performed in the area corresponding to the percutaneous signal. The position of the SLN was accurately determined prior to resection. Percutaneous inguinal SLN detection was successfully achieved in 5 out of 7 selected cases (71 %). A maximum imaging depth of ~20 mm was obtained using a camera exposure time of 80 ms (Fig. 4b; Additional file 1: Video S1).

### Multi-spectral fluorescence detection in endometrial cancer

Independent fluorescence imaging of ICG and MB was performed in two women with endometrial cancer. In both cases, fluorescence co-localisation of the dyes was observed, including sharing of common lymphatics (Fig. 4c), leading to an external iliac SLN irrespective of the injection site and dye (Additional file 2: Video S2). Notably, in the latter case a para-aortic SLN (true positive) was also detected with ICG only, following injection in the uterine fundus (Fig. 4d) but not by MB which was injected in the cervix.

## Discussion

In gynaecological cancers, SLN detection appears to be an attractive and cost-effective alternative to complete lymphadenectomy without jeopardising correct LN staging (21). In an effort to modernise the surgical staging of regional LNs, we conducted a clinical feasibility study to address some of the disadvantages of the existing techniques. We demonstrated the feasibility of using a novel, in-house developed fluorescence imaging system for SLN detection across all the major gynaecological cancers. Surrogate use of NIR fluorescence imaging may improve surgical nodal staging, exclude LN metastases and subsequently reduce the risk of recurrence. Following the learning curve required for a new technique, we reported a cumulative SLN detection rate of 100 % and no false negatives. The total injected volume and concentration of dye(s) was optimised to be 4 ml and 1 mg/ml respectively. No adverse reactions were observed following dye injection.

The main strength of this study was the achievement of the highest SLN detection rate using NIR fluorescence, which, in the context of a clinical trial, outperformed reported conventional methods based on blue dye and/or radioactive tracer [6, 16, 17]. The learning curve indicated a gradual increase in the detection rates following injection and dose optimisation and familiarity with the new technique. Our SLN detection rates agreed with previous studies performed using various NIR fluorescence imaging systems [8, 11, 21]. The detection rates were calculated following exclusion of the initial 33 cases. The drop in detection rate after ~10 cases could be secondary to the later introduction of laparoscopic procedures. In fact, a larger number of laparoscopic cases were required to achieve a satisfactory detection rate, particularly for endometrial cancer. This relatively prolonged learning phase could be possibly justified by the disease heterogeneity (vulval, endometrial and cervical cancers) and the diversity of surgical procedures (open and keyhole surgeries) that were considered in this study, at the obvious advantage of SLN detection using the same imaging modality.

The use of a custom system that can operate as a wide-field device as well as in conjunction with a laparoscope proved to be of significant benefit as fluorescence imaging was performed irrespective of the surgical procedure. Moreover, the system allowed simultaneous bright-field and fluorescence visualisation whilst maintaining reduced size and weight, making it suitable for hand-held operation. Adequate fluorescence sensitivity was reflected by the high intra-operative detection rates during laparoscopy and the percutaneous SLN detection rates in a large proportion of women (71 %) with vulval cancer, during open surgery.

Although most of the procedures (47/49) described in this study were performed following solely ICG injection, MB fluorescence dye was also used in two endometrial cancer cases. Independent fluorescence imaging of two different fluorescence dyes (ICG and MB) was achieved by means of two separate imaging channels available in our instrument. This unique feature, which is not supported in widely used, commercially available NIR fluorescence systems, justified the development and usage of a novel imaging system. Unlike previous studies that used two different imaging modalities to detect SLN involvement from cervical and hysteroscopic injections [19], the use of two fluorescence dyes allowed for a more effective comparison and simultaneous examination of differential lymphatic drainage, as the same imaging modality was adopted.

MB has been successfully used as a blue dye for SLN mapping in patients with endometrial cancer [20]. However, to the best of our knowledge, MB has never been used as a fluorescence dye to detect SLNs. We demonstrated for the first time in a limited number of women (n = 2) that fluorescence SLN visualisation using MB is indeed feasible and provides considerably higher detection sensitivity in comparison to direct viewing. Nevertheless, further experience with fluorescence imaging of MB is required.

The fluorescently detected SLNs successfully predicted the metastatic status of the other LNs in all but four cases, all included in the learning phase. A possible explanation in these cases was the insufficient dose of dye or lack of experience. Pathological LN involvement on the ipsilateral side has also been suggested for SLN detection failure on one side [22]. The highest SLN detection rate was achieved with an ICG concentration of 1 mg/ml and injected volume of 4 ml. This finding was in agreement with a recent meta-analysis on SLN fluorescence biopsy using ICG, which reported a superior detection rate in the sub-group of studies in which the injected volume was ≥2 ml and concentration <5 mg/ml [23].

We showcased extracorporeal visualisation of SLNs in the majority of women with vulval cancer following ICG injection which led to earlier SLN detection times. Such detection proved to be accurate up to a depth of ~20 mm below the skin. As SLNs in vulval cancer are superficially located, NIR fluorescence imaging has strong potential to reduce invasiveness as excision could be guided by transcutaneous fluorescence. However, difficulties were observed in patients with a high BMI (>30) as the available excitation light intensity could not penetrate the greater tissue thickness. This was in agreement with the findings from a similar study on vulval cancer [11].

We reported, for the first time in human, an independent fluorescence detection of ICG and MB in women with endometrial cancer, which revealed an association between injected site and SLN location. To date, there is still widespread controversy on the most appropriate injection practice for SLN detection in endometrial cancer [14]. Given the limitations in the accessibility of peri-tumoural regions, a common practice is to inject the tracer in the cervical region [24]. Another important aspect of SLN detection is the ability to map the para-aortic LNs, which also appears to be highly dependent to the injection location. Using fluorescence imaging at more than a single wavelength, we suggested that cervical injection in endometrial cancer might not be fully representative of the tumour. This was highlighted by the fluorescence detection of a para-aortic SLN, taking up only the dye (ICG) injected in the uterine fundus. Although this finding was previously reported in studies in which women were either divided in two injection groups [25] or by using different imaging modalities [19], our study is the first, in which both sites were concurrently injected in the same woman and the same imaging modality was employed to examine the aberrant lymphatic drainage independently. By this approach, we eliminated any in-between patient variability and differences encountered in the detection sensitivities of radioactive and blue dye methods.

We carried out a comprehensive literature review for SLN detection using NIR fluorescence in gynaecological caners (Table 3). The feasibility of SLN detection was demonstrated without any disparity in the safety profile. In all but one of the studies, the overall SLN detection rate ranged from 64 to 100 % using a variety of imaging devices. Our study, however, is the only one to report the use of the same imaging system, which can be used to standardise how SLN detection is compared across all cancer types. This is desirable when the imaging modality strongly relies on the sensitivity of the instrument.

**Table 3:** Review summary of SLN biopsy in gynaecological cancers using NIR fluorescence

Reference	Cancer type	Surgical procedure	Imaging device	No. of patients	Key results
Furukawa [26]	Cervical	Open	Photodynamic eye (Hamamatsu)	12	At least 1 SLN identified in 83 % of the patients
Crane [27]	Cervical	Open	Custom made prototype	10	Intraoperative detection rate = 64 % relative to total number of ex vivo fluorescent SLNs
Crane [11]	Vulvar	Open	Custom made prototype	10	Detection rates relative to gamma probe: fluorescence = 89.7 %, patent blue = 72.4 %
Van der Vorst [10]	Cervical	Open	Mini-FLARE	9	At least 1 SLN identified in each patient. Optimal ICG:HSA dose: 500 μM
Rossi [28]	Endometrial/cervical	Robotic-assisted laparoscopy	SPY scope (Novadaq)	20	At least 1 SNL identified in 85 % of the cases
Holloway [8]	Endometrial	Robotic-assisted laparoscopy	Da Vinci NIR fluorescence imaging system	35	At least 1 SLN identified in 97 % of the patients with fluorescence and 77 % with calorimetric analysis
Hutteman [9]	Vulvar	Open	Mini-FLARE	9	Detection rates relative to gamma probe: fluorescence = 100 %, patent blue = 71 % Optimal ICG:HSA dose: 750 μM
Schaafsma [29]	Cervical	Open	Mini-FLARE	18	At least 1 SLN identified in 97 % of the patients. No significant difference in signal-to-background ratio between ICG alone and ICG:HSA
Schaafsma [13]	Vulval	Open	Mini-FLARE	24	Detection rates relative to gamma probe: fluorescence = 100 %, patent blue = 77 % No significant difference in detection rate between ICG alone and ICG:HSA
Rossi [12]	Endometrial	Robotic-assisted laparoscopy	SPY^®^ scope (Novadaq)	29	SLN detection rate: 82 % for cervical injection, 33 % for hysteroscopic endometrial injection
Mathéron [18]	Vulval	Open	Photodynamic eye (Hamamatsu)	15	SLN detection rate: 98 % radioactive, 96 % fluorescence, 65 % blue dye
Jewell [21]	Endometrial/cervical	Robotic-assisted laparoscopy	PINPOINT^®^ (Novadaq)	227	SLN detection rate: 95 %. Combined use of ICG and blue dye proved unnecessary
Sinno [30]	Endometrial	Robotic-assisted laparoscopy	PINPOINT^®^ (Novadaq)	71	Fluorescence ICG superior than blue dye (78.9 % vs. 42.4 % bilateral detection rate)

## Conclusion

This study demonstrated the feasibility of SLN mapping in women with vulval, cervical and endometrial cancers using a novel, customised NIR fluorescence imaging device. We showed a 100 % SLN detection rate and maximum specificity and sensitivity following learning phase and dose optimisation. We observed percutaneous SLN fluorescence in patients with vulval cancer during open surgery which resulted in reduced SLN detection times. We employed multiple fluorescence dyes to visualise independent draining lymphatic paths in women with endometrial cancer.

## Additional files

10.1186/s13104-015-1576-z Percutaneous SLN detection in vulval cancer using the imaging system in wide-field mode.
10.1186/s13104-015-1576-z Fluorescence surgical navigation in endometrial cancer using the imaging system in laparoscopic mode and multi-spectral fluorescence detection.

## Abbreviations

ICG: indocyanine green
MB: methylene blue
NIR: near infrared
LN: lymph node
SLN: sentinel lymph node
BMI: body mass index
